# Pituitary Apoplexy: A Rare but Critical Emergency in Neuroendocrinology

**DOI:** 10.7759/cureus.77970

**Published:** 2025-01-25

**Authors:** Mrooj M Almutairi, Ghadeer M Thamer, Khalid F Alharthi, Huda J Ali, Anas E Ahmed

**Affiliations:** 1 College of Medicine, King Abdulaziz University, Jeddah, SAU; 2 College of Medicine, Wenzhou Medical University, Wenzhou, CHN; 3 General Practice, Jazan University, Jazan, SAU

**Keywords:** adenoma, adrenal insufficiency, cranial nerve palsy, headache, hemorrhage, hypopituitarism, infarction, pituitary apoplexy, pituitary gland, visual disturbances

## Abstract

Pituitary apoplexy is a rare and potentially life-threatening condition resulting from a sudden hemorrhage or infarction of the pituitary gland, often within a pre-existing adenoma. This report describes the case of a 45-year-old male patient who presented with an acute onset of a severe headache, visual disturbances, and cranial nerve palsy. Laboratory investigations revealed hypopituitarism with adrenal insufficiency, and magnetic resonance imaging confirmed a hemorrhagic pituitary macroadenoma compressing the optic chiasm. Prompt initiation of corticosteroid therapy and urgent transsphenoidal surgical decompression led to significant clinical improvement, including the resolution of visual and neurological deficits. Histopathological examination confirmed a nonfunctioning pituitary adenoma with extensive hemorrhagic infarction. This case emphasizes the importance of early recognition, timely endocrine management, and surgical intervention in optimizing outcomes for patients with pituitary apoplexy. It also highlights the need for long-term endocrine follow-up due to the high risk of persistent hypopituitarism. The multidisciplinary approach demonstrated here aligns with current evidence-based guidelines and underscores the critical role of collaboration in managing this neuroendocrine emergency.

## Introduction

Pituitary apoplexy is a rare but potentially life-threatening clinical syndrome caused by a sudden hemorrhage or infarction of the pituitary gland, typically within a pre-existing pituitary adenoma. It accounts for less than 10% of all cases of pituitary adenomas and is considered a neuroendocrine emergency [1,2]. The condition often presents with a constellation of symptoms, including severe headache, visual disturbances, ophthalmoplegia, and altered mental status. These symptoms result from the abrupt expansion of the sellar mass, leading to the compression of adjacent structures such as the optic chiasm, cranial nerves, and the hypothalamic-pituitary axis [1-3].

While the exact triggers for pituitary apoplexy are not always identifiable, several precipitating factors have been reported, including anticoagulation therapy, major surgery, head trauma, pregnancy, and systemic illnesses [2-4]. Endocrine dysfunction, particularly adrenal insufficiency, is a common and critical feature of this condition that requires prompt recognition and management to avoid life-threatening complications [1,5].

Diagnosis is typically established through a combination of clinical suspicion, hormonal evaluation, and imaging studies, particularly magnetic resonance imaging (MRI). Treatment involves the rapid stabilization of the patient’s hemodynamic and endocrine status, followed by neurosurgical intervention when indicated [2-4]. This report describes a case of pituitary apoplexy, emphasizing the importance of early diagnosis and multidisciplinary management in optimizing patient outcomes.

## Case presentation

A 45-year-old male patient with no significant past medical history presented to the emergency department with a sudden onset of severe headache, which he described as the worst headache of his life. The headache was located predominantly in the frontal and retro-orbital regions and was associated with nausea, vomiting, and photophobia. He reported a sudden episode of blurred vision and diplopia, which occurred concurrently with the onset of the headache. There was no history of fever, head trauma, or recent illness. The patient denied any significant weight changes, polyuria, polydipsia, or prior symptoms suggestive of an endocrine dysfunction. He had no known family history of pituitary or endocrine disorders and was not on any medications.

On arrival, the patient was alert but appeared in significant distress due to the pain. His vital signs were stable, with a blood pressure of 128/76 mmHg, heart rate of 82 beats per minute, and afebrile temperature. Neurological examination revealed a left-sided partial third cranial nerve palsy with ptosis and impaired adduction of the left eye but preserved pupil response to light. Visual field testing demonstrated a bitemporal hemianopia, consistent with the compression of the optic chiasm. The remainder of the cranial nerve examination was unremarkable and no focal neurological deficits were observed. The cardiovascular, respiratory, and abdominal examinations were within normal limits.

Initial laboratory investigations revealed a normal complete blood count, basic metabolic panel, and coagulation profile. However, serum cortisol levels were notably reduced at 3.2 µg/dL (reference range: 6.2-19.4 µg/dL), suggestive of adrenal insufficiency. Thyroid function tests showed a free T4 level of 0.6 ng/dL (reference range: 0.8-2.0 ng/dL) and a TSH level of 0.3 µIU/mL (reference range: 0.4-4.0 µIU/mL), indicating central hypothyroidism. Prolactin, luteinizing hormone, follicle-stimulating hormone, and testosterone levels were also evaluated and were found to be within the lower-normal range, raising concern for hypopituitarism. Serum sodium was mildly low at 131 mEq/L (reference range: 135-145 mEq/L), indicative of a possible secondary adrenal insufficiency.

**Figure 1:**
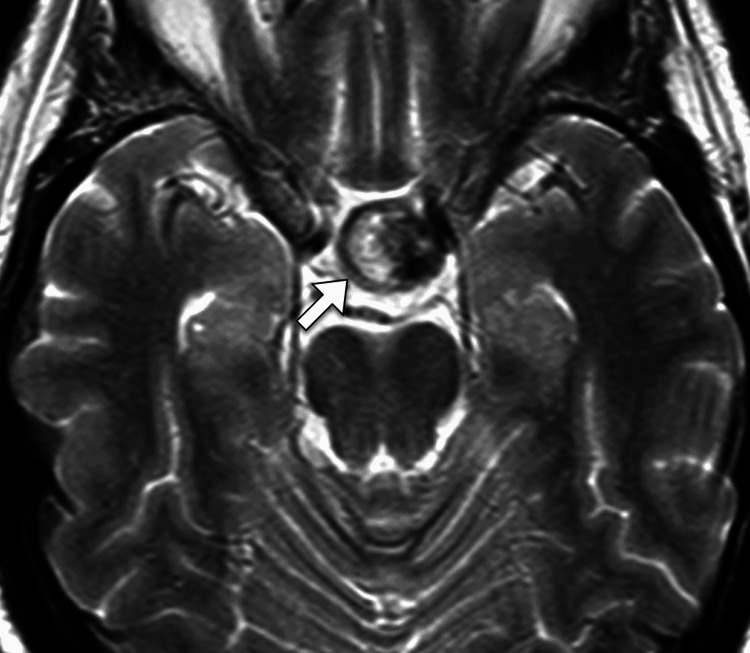
Axial T2-weighted magnetic resonance image of the brain demonstrating a mixed signal intensity sellar lesion with hypointense components (arrow)

Given the acute presentation and clinical suspicion of pituitary apoplexy, urgent imaging studies were performed. A non-contrast computed tomography scan of the head revealed a sellar mass with mixed-density components, suggestive of a hemorrhage. Subsequent MRI of the brain with contrast demonstrated a 2.2 cm sellar and suprasellar lesion consistent with a pituitary macroadenoma (Figure 1).

The lesion showed evidence of intralesional hemorrhage with compression of the optic chiasm and displacement of the pituitary stalk. There was no evidence of a cavernous sinus invasion or hydrocephalus. These imaging findings, combined with the clinical presentation, confirmed the diagnosis of pituitary apoplexy.

The patient was promptly started on high-dose intravenous hydrocortisone to address adrenal insufficiency. He was also administered levothyroxine after the stabilization of cortisol levels to manage central hypothyroidism. Neurosurgery was consulted, and after a discussion of the risks and benefits, we made a decision to proceed with an urgent transsphenoidal surgical decompression to relieve the optic chiasm compression and prevent further neurological deterioration.

The patient underwent successful transsphenoidal surgery, during which the hemorrhagic and necrotic pituitary adenoma was resected. Postoperative recovery was uneventful, and the patient reported significant improvement in the headache and visual symptoms within 48 hours. A repeat neurological examination showed resolution of the third cranial nerve palsy, and follow-up visual field testing confirmed an improvement in the bitemporal hemianopia. Histopathological examination of the resected tissue confirmed the diagnosis of a nonfunctioning pituitary adenoma with extensive hemorrhagic infarction.

During his hospital stay, the patient’s endocrine function was closely monitored. He required ongoing hydrocortisone replacement therapy and was discharged on a maintenance dose. Endocrinology follow-up was arranged to reassess his hormone levels and adjust replacement therapy as needed. At the six-week follow-up visit, the patient continued to show marked clinical improvement, with resolution of the hyponatremia and stabilization of the thyroid function on levothyroxine. He was counseled on the potential for long-term hypopituitarism and the need for lifelong endocrine follow-up.

## Discussion

The present case of pituitary apoplexy illustrates the complexity and urgency associated with this neuroendocrine emergency, highlighting the interplay of clinical presentation, diagnostic strategies, and therapeutic interventions. The acute onset of symptoms in this patient, characterized by severe headache, visual disturbances, and cranial nerve palsy, underscores the classic presentation of pituitary apoplexy. Such cases demand prompt recognition and intervention to mitigate the risk of permanent neurological and endocrine dysfunction [1,3].

Pituitary apoplexy occurs most commonly in the setting of a pre-existing pituitary adenoma, as seen in this case. The pathophysiology involves a sudden hemorrhage or infarction within the adenoma, leading to a rapid expansion of the sellar mass [2-4]. This, in turn, causes a compression of the adjacent structures, such as the optic chiasm and cranial nerves, as well as a disruption of the pituitary gland’s hormonal output. While the patient’s initial presentation with bitemporal hemianopia and third cranial nerve palsy was consistent with compression-related symptoms, the associated hypopituitarism, including adrenal insufficiency and central hypothyroidism, reflected the functional impact of pituitary damage [1,4].

The diagnostic approach in this case aligned with established best practices, emphasizing the importance of imaging and hormonal evaluation. An MRI remains the gold standard for detecting pituitary apoplexy as it provides a detailed visualization of the sellar region and the associated hemorrhagic changes [2,3]. The imaging findings of a sellar and suprasellar lesion with the intralesional hemorrhage in this patient were pathognomonic, correlating well with the clinical picture. Early biochemical assessment, including cortisol and thyroid function tests, was crucial in identifying life-threatening endocrine deficiencies and guiding the initial management [1-3].

The management of pituitary apoplexy necessitates a multidisciplinary approach, combining endocrinological and neurosurgical expertise [1,2]. In this case, the rapid initiation of hydrocortisone replacement therapy was critical for addressing the adrenal insufficiency and preventing an adrenal crisis. The decision to proceed with transsphenoidal surgical decompression reflects current consensus guidelines, which recommend surgery for patients with significant visual or neurological impairment. The patient’s favorable postoperative outcomes, including improvement in visual fields and resolution of cranial nerve palsy, highlight the efficacy of timely surgical intervention.

A comparison with the literature reveals that this case aligns with the typical demographic and clinical features of pituitary apoplexy. The condition predominantly affects middle-aged individuals, with a slight male predominance. While the exact precipitating factor in this patient remained unclear, other studies have identified triggers such as anticoagulation, major surgery, and systemic infections. Interestingly, some reports have also highlighted spontaneous cases, emphasizing the unpredictable nature of this condition [5,6].

Some general aspects of pituitary apoplexy warrant further discussion. Despite its rarity, the condition’s potential for severe morbidity underscores the need for heightened clinical vigilance, especially in patients with known pituitary adenomas. The role of conservative management in select cases, typically those with mild or stable symptoms, remains a topic of ongoing debate. However, surgical decompression is widely regarded as the definitive treatment for patients with progressive visual or neurological deficits.

An endocrine follow-up is a critical aspect of post-apoplexy care, as long-term hypopituitarism is common even in surgically treated patients. This case reinforces the importance of regular hormonal assessments and individualized replacement therapy to optimize patient outcomes. Additionally, it highlights the need for patient education regarding the potential for recurrence and the importance of adherence to follow-up care.

## Conclusions

Pituitary apoplexy remains a rare but critical medical emergency that necessitates timely diagnosis and intervention to prevent lasting neurological and endocrine complications. This case underscores the importance of maintaining a high index of suspicion in patients presenting with acute headache, visual deficits, and cranial nerve dysfunction, particularly in the context of known pituitary adenomas. The rapid initiation of appropriate medical therapy, including corticosteroid replacement, combined with surgical decompression when indicated, can lead to significant recovery and improved patient outcomes. Long-term management requires vigilant endocrine follow-up to address persistent hypopituitarism and ensure optimal quality of life. This case highlights the need for a multidisciplinary approach and adherence to evidence-based protocols in managing this complex condition.
